# Antiproliferative Action of Conjugated Linoleic Acid on Human MCF-7 Breast Cancer Cells Mediated by Enhancement of Gap Junctional Intercellular Communication through Inactivation of NF-**κ**B

**DOI:** 10.1155/2013/429393

**Published:** 2013-11-25

**Authors:** Md. Abdur Rakib, Won Sup Lee, Gon Sup Kim, Jae Hee Han, Jeong Ok Kim, Yeong Lae Ha

**Affiliations:** ^1^Division of Applied Life Sciences (BK21 Plus), Graduate School, and Institute of Agriculture & Life Science, Gyeongsang National University, Jinju 660-701, Republic of Korea; ^2^Department of Biochemistry and Molecular Biology, Faculty of Science, University of Rajshahi, Rajshahi-6205, Bangladesh; ^3^Department of Internal Medicine and Institute of Health Sciences, Gyeongsang National University, School of Medicine, Jinju 660-702, Republic of Korea; ^4^Laboratory of Biochemistry (BK21 Plus), School of Veterinary Medicine, Gyeongsang National University, Jinju 660-701, Republic of Korea; ^5^Department of Physiology and Institute of Health Sciences, Gyeongsang National University, School of Medicine, Jinju 660-702, Republic of Korea; ^6^HK Biotech Co., Ltd., Jinju 660-844, Republic of Korea

## Abstract

The major conjugated linoleic acid (CLA) isomers, *c*9,*t*11-CLA and *t*10,*c*12-CLA, have anticancer effects; however, the exact mechanisms underlying these effects are unknown. Evidence suggests that reversal of reduced gap junctional intercellular communication (GJIC) in cancer cells inhibits cell growth and induces cell death. Hence, we determined that CLA isomers enhance GJIC in human MCF-7 breast cancer cells and investigated the underlying molecular mechanisms. The CLA isomers significantly enhanced GJIC of MCF-7 cells at 40 **μ**M concentration, whereas CLA inhibited cell growth and induced caspase-dependent apoptosis. CLA increased connexin43 (Cx43) expression both at the transcriptional and translational levels. CLA inhibited nuclear factor-**κ**B (NF-**κ**B) activity and enhanced reactive oxygen species (ROS) generation. No significant difference was observed in the efficacy of *c*9,*t*11-CLA and *t*10,*c*12-CLA. These results suggest that the anticancer effect of CLA is associated with upregulation of GJIC mediated by enhanced Cx43 expression through inactivation of NF-**κ**B and generation of ROS in MCF-7 cells.

## 1. Introduction

Conjugated linoleic acid (CLA) is comprised of positional and geometric isomers of octadecadienoic acid (C18:2) with a conjugated double bond [1]. CLA has several health benefits *in vivo*, including anticarcinogenesis, antiatherogenesis, antiobesity, and modulation of immune function [1–5]. Numerous anticancer and anticarcinogenic effects of CLA and individual CLA isomers have been reported. CLA inhibits 7,12-dimethylbenz[*a*]anthracene-induced mouse skin carcinogenesis, benzo*(a)*pyrene-induced mouse forestomach tumorigenesis, 2-amino-3,4-dimethylimidazo[4,5-*f*] quinoline-induced rat colonic carcinogenesis, and *N*-methyl-*N*-nitrosourea-induced rat mammary carcinogenesis [1, 2, 6, 7]. Individual CLA isomers also show anticancer effects by inducing apoptosis in animals and cancer cell lines [7–9], but their mechanisms of action are still unknown. 

Gap junctions are transmembrane channels that connect the cytoplasm of neighboring cells composed of two hemichannels and each consists of six connexin proteins [10]. Intercellular communication through gap junctions plays an important role in maintaining tissue homeostasis by allowing the passage of small cytoplasmic molecules and ions (<1 kD), such as cyclic AMP, diacylglycerol, inositol triphosphate, and Ca^+2^ by the process known as gap junctional intercellular communication (GJIC) [11]. GJIC has been postulated to regulate cell proliferation, differentiation, apoptosis, and adaptive responses of differentiated cells [11, 12]. Tumor promoters downregulate GJIC in carcinogenesis [13, 14], and GJIC is suppressed in most cancer cells compared to normal cells [15, 16]. In addition, growth factors and several oncogenes inhibit GJIC [17], and cancer drugs reverse downregulation of GJIC [18–21]. However, nothing is known regarding whether CLA isomers can reverse suppressed GJIC in cancer cells.

Up to now, three connexin proteins have been identified in human breast tissue, including Cx43, Cx26, and Cx32 [22–24]. Cx43 is the most widely studied of these connexin proteins because of its high expression in most cells [22]. Mutations or deficiencies in connexin genes are related to several diseases including cancer; thus, transfection of connexin genes into GJIC-deficient tumor cells restores the reduced GJIC and suppresses tumor growth [25–28]; therefore, connexin genes are related to restoration of GJIC in cancer cells. We previously demonstrated that CLA isomers prevent 12-*O-*tetradecanoylphorbol-13-acetate (TPA) induced downregulation of GJIC in non-tumorigenic mammary epithelial MCF-10A cells by inhibiting Cx43 phosphorylation [13, 29].

Therefore, we hypothesized that CLA isomers enhance GJIC in cancer cells by inducing Cx43 gene expression and that the enhancement of GJIC is related to anticancer effects. Here, we investigated the anticancer effects of major CLA isomers, *c*9,*t*11-CLA and *t*10,*c*12-CLA, on human MCF-7 breast cancer cells and the underlying molecular mechanisms.

## 2. Materials and Methods 

### 2.1. Reagents

Roswell Park Memorial Institute (RPMI) 1640 medium, fetal bovine serum (FBS), and penicillin-streptomycin were obtained from Gibco BRL (Rockville, MD, USA). Bovine serum albumin (BSA), 3-(4,5-dimethylthiazol-2-yl)-2,5-diphenyltetrazolium bromide (MTT), and phosphate-buffered saline (PBS) were purchased from Amresco (Solon, OH, USA). *α*-Tocopherol phosphate, L-ascorbic acid, Lucifer yellow dilithium salt (LY), 2′,7′-dihydrodichlorofluorescein diacetate (DCFH-DA), linoleic acid (LA, 99%), sodium selenite, transferrin, phenylmethanesulfonyl fluoride (PMSF), ribonuclease A, propidium iodide (PI), 0.25% trypsin containing 2 mM EDTA, and dimethyl sulfoxide (DMSO) were purchased from Sigma-Aldrich (St. Louis, MO, USA). Trizol reagent was purchased from Invitrogen (Carlsbad, CA, USA). An Omniscript Reverse Transcriptase kit was purchased from Qiagen Inc. (Valencia, CA, USA). Super-Therm DNA polymerase was obtained from JMR (Side Cup, Kent, UK). Radio immune precipitation assay (RIPA) buffer was purchased from Cell Signaling Technology (Danvers, MA, USA). Mouse monoclonal anti-Cx43 IgM, goat-anti-mouse IgM-FITC, goat-anti-mouse IgM, and mouse monoclonal anti-NF-*κ*B IgG were purchased from Santa Cruz Biotechnology (Santa Cruz, CA, USA). Rabbit polyclonal caspase-3 antibody was purchased from Delta Biolabs (Campbell, CA, USA). Mouse monoclonal anti-**β**-actin was purchased from Sigma-Aldrich. Cx43 gene of primers for 5′-ACATCAGGTGGACTGTTTCCT-3′ (sense) and 5′-ACGACTGCTGGCTCTGCTT-3′ (antisense) and **β**-actin primers 5′-CGTGACATCAAGGAGAAGCT-3′ (sense) and 5′-ATCCACATCTGCTGGAAGGT-3′ (antisense) were obtained from ABgene (Epsom, UK). All other reagents used in this study were of analytical grade.

### 2.2. Preparations of CLA Isomers

The *c*9,*t*11-CLA and *t*10,*c*12-CLA isomers were isolated from synthetic CLA methyl ester by low-temperature crystallization at −68 and −71°C in conjunction with urea treatment [30]. The purity of the CLA isomers was 94.5% for *c*9,*t*11-CLA and 98.5% for *t*10,*c*12-CLA when analyzed by gas chromatography [30]. Each CLA isomer or LA was complexed with fatty acid-free BSA according to the method described by van Greevenbroek et al. [31].

### 2.3. Cell Culture and Sample Treatments

The MCF-7 human breast cancer cells (Korean Cell Line Bank, Seoul, Republic of Korea) were cultured in RPMI 1640 medium supplemented with 10% FBS and penicillin-streptomycin in a humidified atmosphere with 5% CO_2_ at 37°C as described previously [8]. The cells were grown in Nunc culture dishes (35 mm, i.d., Rochester, NY, USA) to a confluency of 80% and trypsinized with 0.25% trypsin containing 2 mM EDTA to disperse the cells. The cells were subsequently collected by centrifugation (1000 g for 10 min) and resuspended in fresh culture media.

The cells were seeded either into a 96-well culture plate (Nunc) at 1 × 10^4^ cells per well for the cell proliferation test using the MTT assay or Nunc culture dish (10 mm, i.d.) at 1 × 10^5^ cells for other assays. After 24 h incubation, the cells were serum starved by an additional 24 h incubation in RPMI 1640 medium supplemented with 5 *μ*g/mL transferrin, 5 ng/mL sodium selenite, and 0.1 mg/mL BSA.

Two experiments were conducted for the cytotoxicity assay. In the first experiment, the serum-starved MCF-7 cells were incubated with *c*9,*t*11-CLA (0–40 *μ*M) in RPMI 1640 medium, supplemented with ascorbic acid (50 ng/mL) and *α*-tocopherol phosphate (20 ng/mL) for 72 h to determine the optimal concentration and incubation time. In the second experiment, the cytotoxicity of *c*9,*t*11-CLA was compared to that of *t*10,*c*12-CLA and LA for 48 h using a 40 *μ*M concentration. For the other experiments, the serum-starved cells were incubated with 40 *μ*M *c*9,*t*11-CLA, *t*10,*c*12-CLA, and LA for 48 h. After incubation with the CLA isomers, the cells were trypsinized with 0.25% trypsin containing 2 mM EDTA and collected by centrifugation (1000 g for 10 min). These cells were washed with PBS and collected again by centrifugation before assay. Fresh medium was replaced in every 48 h. The exponentially growing cells were used throughout the experiments.

### 2.4. Cell Viability Assay

MCF-7 cell proliferation was measured using the MTT assay as described previously [29]. Briefly, the cells treated with the CLA isomers were exposed to MTT solution (5 mg/mL of PBS) for 4 h. The MTT solution was removed, and 200 *μ*L DMSO was added to each well and mixed to dissolve the MTT formazan crystals formed by the viable cells. The absorbance of each well was measured at 570 nm using an Anthos 2020 microplate reader (Wals, Austria). Data are represented as relative cell viability against the cells at time 0.

### 2.5. Flow Cytometry Analysis

The cell cycle was analyzed as described previously [32]. Briefly, the cells treated with the CLA isomers were collected in 1 mL ice-cold PBS and fixed with 4 mL 70% ethanol for 30 min at 4°C. The fixed cells were washed with PBS and then incubated with 500 *μ*L PI solution (1 mg/mL of RNAase, 50 *μ*g/mL of PI, and 0.1% TritonX-100) for 20 min at 37°C. Flow cytometric analysis was performed using a BD FACSCalibur Flow Cytometer (BD Science, San Jose, CA, USA) equipped with CellQuest Pro Software (BD Science).

### 2.6. Hoechst 33258 Staining

The cells treated with the CLA isomers were fixed with 4% paraformaldehyde in PBS for 10 min and permeabilized in PBS containing 0.1% Triton X-100 for 10 min, after which the nuclei were stained with Hoechst dye (10 *μ*g/mL) for 10 min as described previously [8, 9]. Finally, the cells were observed under an Olympus IX70 inverted fluorescent microscope (Okaya, Japan).

### 2.7. GJIC Assay

GJIC of cells treated with CLA isomers was measured using the scrape loading/dye transfer (SL/DT) technique as previously described [29]. Briefly, cells were treated with 0.05% LY solution and scraped with a surgical steel scalpel blade at low light intensity. After 3 min of incubation (5% CO_2_, 37°C), the cells were washed four times with 2 mL PBS and fixed with 4% paraformaldehyde. The number of dye-communicating cells perpendicular to the scraped-line was counted under an Olympus IX70 inverted fluorescent microscope.

### 2.8. RNA Extraction and Reverse Transcription Polymerase Chain Reaction (RT-PCR) for Cx43 Gene Expression

Total RNA of cells treated with the CLA isomers was isolated using Trizol reagent according to the method described by the manufacturer. RT-PCR analysis was performed as described previously [33]. cDNA was synthesized from total RNA using an Omniscript Reverse Transcriptase kit according to the manufacturer's instructions. The Cx43 gene was amplified by RT-PCR using 5′-ACATCAGGTGGACTGTTTCCT-3′ (sense) and 5′-ACGACTGCTGGCTCTGCTT-3′ (antisense) as primers in a 50 *μ*L final volume containing Super-Therm DNA polymerase, 1.5 mM MgCl_2_, and 2 mM dNTP for 30 cycles (denaturation at 94°C for 30 seconds, annealing at 50°C for 1 min, and extension at 72°C for 1 min, followed by final incubation at 72°C for 7 min). **β**-actin primers 5′-CGTGACATCAAGGAGAAGCT-3′ (sense) and 5′-ATCCACATCTGCTGGAAGGT-3′ (antisense) were used as the control under the same PCR conditions. PCR products were analyzed by electrophoresis on 1% agarose gels.

### 2.9. Western Blot Analysis

Western blot analysis was performed as described previously [29]. Cells treated with CLA isomers were lysed at 4°C by shaking for 15 min followed by homogenization with RIPA buffer. After centrifugation at 13,000 g for 15 min, the supernatant was collected and stored at −70°C until use. Protein concentration was determined using the Bradford reagent (Hercules, CA, USA). Proteins were separated by 12.5% SDS-polyacrylamide gel electrophoresis, and Western blot analysis of Cx43, caspase-3, and NF-*κ*B was performed according to the manufacturer's instructions. Protein bands were detected using an Enhanced Chemiluminescence Detection kit (Thermo Scientific, Rockford, IL, USA).

### 2.10. Preparation of Nuclear Protein for Western Blotting of NF-*κ*B

Proteins in the nuclear fraction of cells were isolated as described previously [34]. Cells treated with CLA isomers were incubated for 20 min at 48°C in a buffer containing 20 mM *N*-(2-hydroxyethyl)-piperazine-*N*′-(2-ethanesulfonic acid) (HEPES) KOH (pH 7.9), 25% glycerol, 420 mM NaCl, 1.5 mM MgCl_2_, 0.2 mM EDTA, 0.5 mM dichlorodiphenyltrichloroethane (DTT), and 0.2 mM PMSF. Cell nuclei were collected as a pellet by centrifugation at 14,000 g for 5 min at 48°C. Nuclear proteins were extracted at 4°C by gently resuspending the nuclear pellets in a buffer solution containing 20 mM Tris (pH 7.5), 20% glycerol, 1.5 mM MgCl_2_, 420 mM NaCl, 0.2 mM EDTA, and 0.1% Triton X-100, followed by a 1 h incubation at 4°C with occasional vortexing. After centrifugation at 14,000 g for 15 min at 4°C, the supernatant, which contained the nuclear protein fraction, was collected. Protein concentration was determined using the Bradford reagent.

### 2.11. Immunocytochemistry Assay of Cx43 Protein

The cells treated with CLA isomers were seeded and cultured on chamber slides for immunocytochemistry analysis as described previously [35]. Cells were fixed with 4% paraformaldehyde on chamber slides. The fixed cells were permeabilized with 0.5% Triton X-100 in PBS and incubated with a blocking solution (PBS containing 2% BSA) at room temperature for 30 min. The cells were then incubated with anti-connexin 43 IgM as a primary antibody and FITC-conjugated goat-anti-mouse IgM as the secondary antibody. Fluorescent staining of Cx43 was imaged using an Olympus IX70 inverted fluorescent microscope.

### 2.12. Intracellular Reactive Oxygen Species (ROS) Assay

Intracellular ROS positive cells were determined as described previously [36]. Cells treated with CLA isomers were incubated with 50 *μ*M DCFH-DA for 30 min at 37°C in the dark. After the incubation, the cells were washed with Locke's buffer (154 mM NaCl, 25 mM KCl, 2.3 mM CaCl_2_, 3.6 mM NaHCO_3_, 8.6 mM HEPES, and 5.6 mM glucose, pH 7.4). The number of ROS positive cells was measured using an Olympus FV-1000 confocal microscope equipped with an argon laser at 485 nm excitation and 530 nm emission wavelengths.

### 2.13. Statistical Analysis

Data are presented as means ± standard deviations and analyzed using one-way analysis of variance followed by Duncan's multiple range test. Differences were considered significant at *P* < 0.05.

## 3. Results

### 3.1. Growth Inhibition of MCF-7 Cells

**Figure 1:**
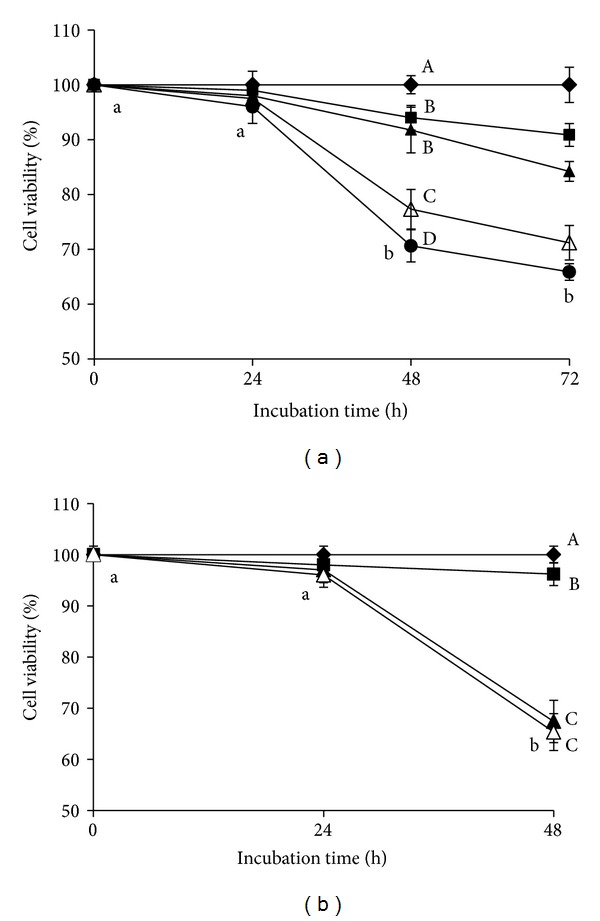
Viability of MCF-7 cells treated with CLA isomers. (a) Cells treated with 5 *μ*M (■), 10 *μ*M (▲), 20 *μ*M (∆), and 40 *μ*M (●) concentrations of *c*9,*t*11-CLA and control (♦) for 72 h. (b) Cells treated with 40 *μ*M *c*9,*t*11-CLA (▲), *t*10,*c*12 -CLA (∆), and LA (■) and control cells (♦) for 48 h. Values are expressed as means ± standard deviations (*n* = 3). Means with different lower case letters or with different upper case letters on different concentration lines at the same incubation time are significantly different at *P* < 0.05 by Duncan's multiple-range test.

First, we determined the optimal concentration of *c*9,*t*11-CLA that showed an anticancer effect on MCF-7 cells. The MTT test was performed on cells grown at various concentrations (5–40 *μ*M) of *c*9,*t*11-CLA for 72 h (Figure 1(a)). The growth inhibitory effect of *c*9,*t*11-CLA was dose and time dependent. At 40 *μ*M, cell viability was 70.6 and 65.9% at 48 and 72 h, respectively (*P* < 0.05).

The inhibitory efficacy of *c*9,*t*11-CLA was compared to that of either *t*10,*c*12-CLA or LA at a concentration of 40 *μ*M for 48 h (Figure 1(b)). *t*10,*c*12-CLA and LA significantly inhibited cell growth by 32.6% and 3.8%, respectively, as compared to that in the control. The potency of *c*9,*t*11-CLA and *t*10,*c*12-CLA was similar. Hence, 40 *μ*M *c*9,*t*11-CLA, *t*10,*c*12-CLA, and LA for 48 h incubation were selected as the optimal conditions for subsequent experiments.

### 3.2. Induction of Apoptosis in MCF-7 Cells

**Figure 2:**
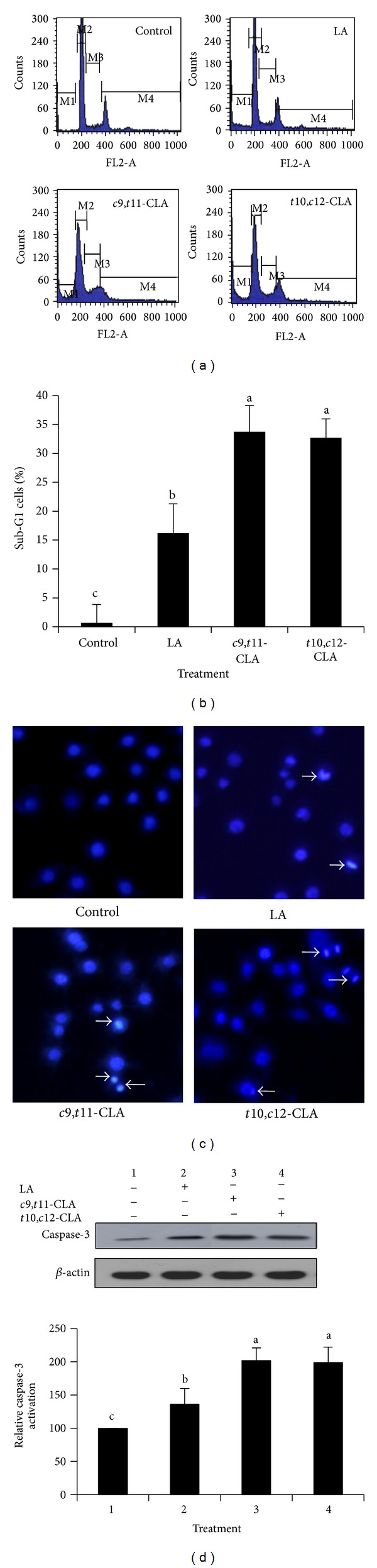
Induction of apoptosis in MCF-7 cells treated with 40 *μ*M *c*9,*t*11-CLA, *t*10,*c*12-CLA, and LA for 48 h. (a) Histograms of cells stained with propidium iodide and analyzed by flow cytometry. The peak area of M1, M2, M3, and M4 represents the cell population of Sub-G1, G0/G1, S, and G2/M phases, respectively. (b) Quantification of Sub-G1 cell population of (a), which is indicative of apoptosis. (c) Hoechst 33258 staining followed by fluorescence microscopic analysis of cells; arrows indicate fragmented or condensed nuclei. (d) Western blot analyses of the key executor enzyme of apoptotic cell death, activated caspase-3. The band intensities relative to control cells were quantified. Values are expressed as means ± standard deviations (*n* = 3). Means with different lowercase letters are significantly different at *P* < 0.05 by Duncan's multiple-range test.

We evaluated whether growth inhibition was related to apoptosis of the cells. The effect of *c*9,*t*11-CLA and *t*10,*c*12-CLA isomers on the cell cycle and apoptotic parameters was evaluated in MCF-7 cells (Figure 2(a)). The cell cycle analysis revealed that *c*9,*t*11-CLA and *t*10,*c*12-CLA significantly increased the percentage of cells in the sub-G1 phase to 35% and 33.6%, respectively, which was an indicator of DNA fragmentation due to cell death (Figure 2(b)). No significant difference in the sub-G1 phase cell population was found between cells treated with the *c*9,*t*11-CLA and *t*10,*c*12-CLA isomers.

To determine whether cell death was related to apoptosis, we further performed Hoechst 33258 staining (Figure 2(c)). As a result, cell shrinkage and nuclear condensation were visible in cells treated with *c*9,*t*11-CLA and *t*10,*c*12-CLA, which indicated apoptosis. To verify whether the apoptosis was caspase dependent, we assessed the level of caspase-3 protein, a key executionary protease, in the process. Western blotting revealed that the *c*9,*t*11-CLA and *t*10,*c*12-CLA isomers significantly increased caspase-3 levels (Figure 2(d)). Taken together, these results indicate that *c*9,*t*11-CLA and *t*10,*c*12-CLA induced cell death by inducing apoptosis.

### 3.3. Enhancement of GJIC in MCF-7 Cells

**Figure 3:**
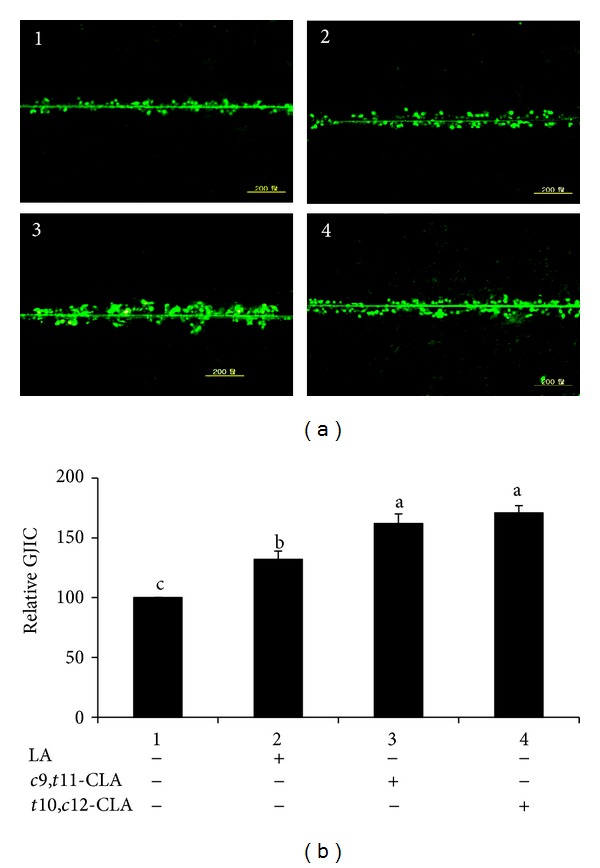
Gap junctional intercellular communication (GJIC) in MCF-7 cells measured by scrape-loading/dye-transfer (SL/DT) technique. MCF-7 cells treated with 40 *μ*M *c*9,*t*11-CLA, *t*10,*c*12-CLA, and LA for 48 h. (a) Representative images of GJIC. (b) Quantification of data (a). Values are expressed as means ± standard deviations (*n* = 3). Means with different lowercase letters are significantly different at *P* < 0.05 by Duncan's multiple-range test.

Next, we determined whether the CLA isomers could reverse the reduced GJIC of MCF-7 cells. We evaluated the status of GJIC in MCF-7 cells treated with *c*9,*t*11-CLA, *t*10,*c*12-CLA, and LA (Figures 3(a) and 3(b)). CLA isomers and LA increased GJIC, relative to control, but the efficacy of the *c*9,*t*11-CLA and *t*10,*c*12-CLA isomers was similar and greater than that of LA. This finding suggests that the inhibition of cell growth might be associated with increased GJIC.

### 3.4. Increased Cx43 Gene Expression in MCF-7 Cells

**Figure 4:**
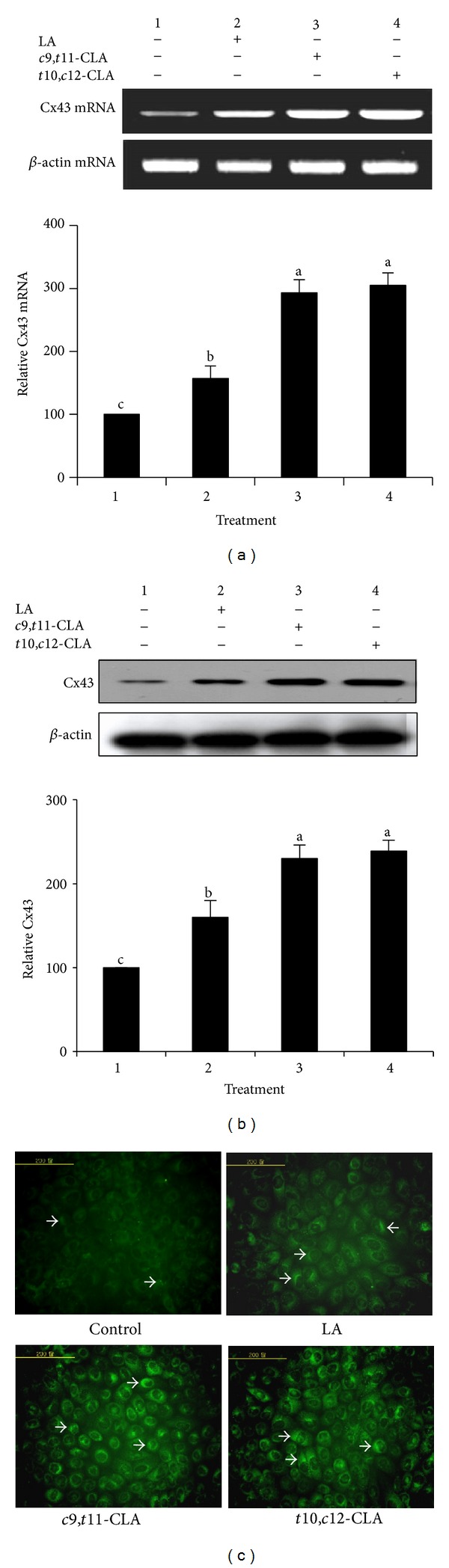
Expression of Cx43 gene in MCF-7 cells treated with 40 *μ*M *c*9,*t*11-CLA, *t*10,*c*12-CLA, and LA for 48 h. (a) Reverse transcription polymerase chain reaction of Cx43 mRNA. (b) Western blot analyses of the Cx43 protein. (c) Representative images of Cx43 expression and its distribution in the plasma membrane of cells, and arrows indicate Cx43 protein expression. Band intensities relative to control cells were quantified. Values are expressed as means ± standard deviations (*n* = 3). Means with different lowercase letters are significantly different at *P* < 0.05 by Duncan's multiple-range test.

Cx43 is a major protein in the gap junction channel that regulates GJIC. Previous results have shown that some chemical compounds upregulate GJIC by upregulating Cx43 expression in human cancer cells [32, 37, 38]. Thus, we assessed the expression levels of the Cx43 gene at the transcriptional and translational levels to investigate the mechanism of GJIC restoration by the CLA isomers (Figure 4). The CLA isomers upregulated the expression of the Cx43 gene at the transcriptional (Figure 4(a)) and translational levels (Figures 4(b) and 4(c)). Both *c*9,*t*11-CLA and *t*10,*c*12-CLA significantly enhanced the level of Cx43 mRNA in MCF-7 cells. No significant difference was observed in the upregulated expression of Cx43 mRNA between *c*9,*t*11-CLA and *t*10,*c*12-CLA. A similar pattern was observed for Cx43 protein expression. These results indicate that both the *c*9,*t*11-CLA and *t*10,*c*12-CLA isomers equally enhanced Cx43 expression.

Immunofluorescence staining was performed to determine the distribution and expression levels of Cx43 in the cell membranes and to further validate the influence of the CLA isomers on Cx43 expression (Figure 4(c)). Compared to the control and LA-treated cells exhibiting only limited expression of Cx43, the cells treated with *c*9,*t*11-CLA and *t*10,*c*12-CLA showed an increased level and wider distribution of the Cx43 protein. The increased immunostaining involved both the extent and size of fluorescent zones, suggesting a possible up-regulation in the number and size of gap junctions.

### 3.5. Generation of Intracellular ROS in MCF-7 Cells

**Figure 5:**
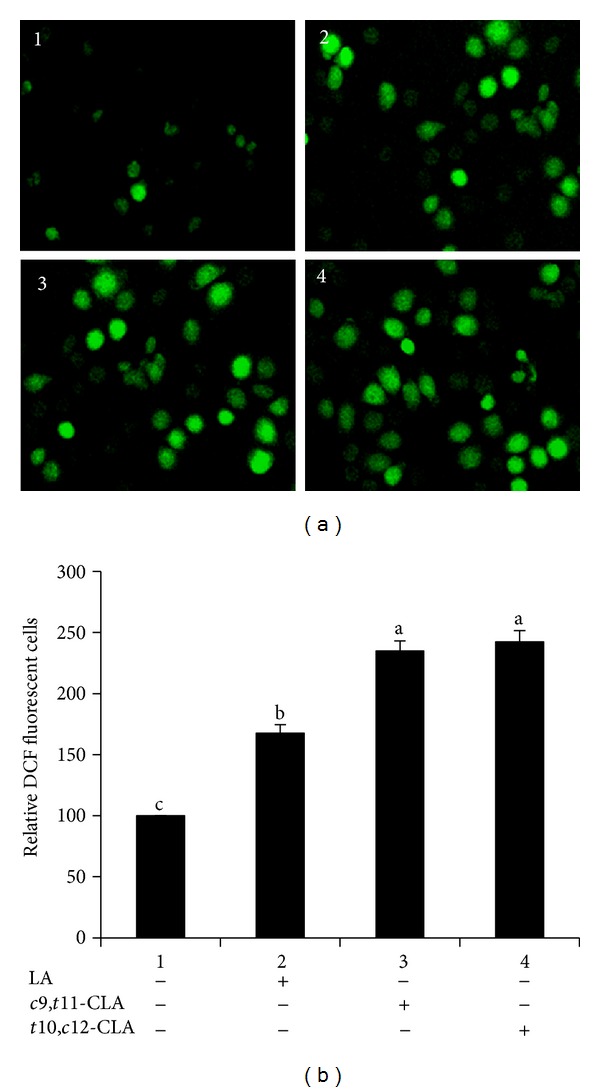
ROS generation in MCF-7 cells treated with 40 *μ*M *c*9,*t*11-CLA, *t*10,*c*12-CLA, and LA for 48 h. (a) Representative images of cells with ROS. (b) Relative DCF-fluorescent MCF-7 cells. Values are expressed as means ± standard deviations (*n* = 3). Means with different lowercase letters are significantly different at *P* < 0.05 by Duncan's multiple-range test.

There is evidence that elevated ROS depolarizes the cell membrane and opens connexin hemichannels to increase connexin gene expression, resulting in increased cell death [39]. Hence, we evaluated intracellular ROS generation in MCF-7 cells after CLA treatment. The effect of *c*9,*t*11-CLA and *t*10,*c*12-CLA isomers on ROS generation was measured by DCFH-DA dye method at 40 *μ*M concentration for 48 h (Figures 5(a) and 5(b)). The levels of DCF fluorescence in the cells treated with *c*9,*t*11-CLA and *t*10,*c*12-CLA increased significantly relative to the control and LA-treated cells. No significant difference in ROS accumulation was found between cells treated with *c*9,*t*11-CLA and *t*10,*c*12-CLA. These results indicate that the CLA isomers induced the formation of ROS.

### 3.6. Induction of NF-*κ*B Inactivation in MCF-7 Cells

**Figure 6:**
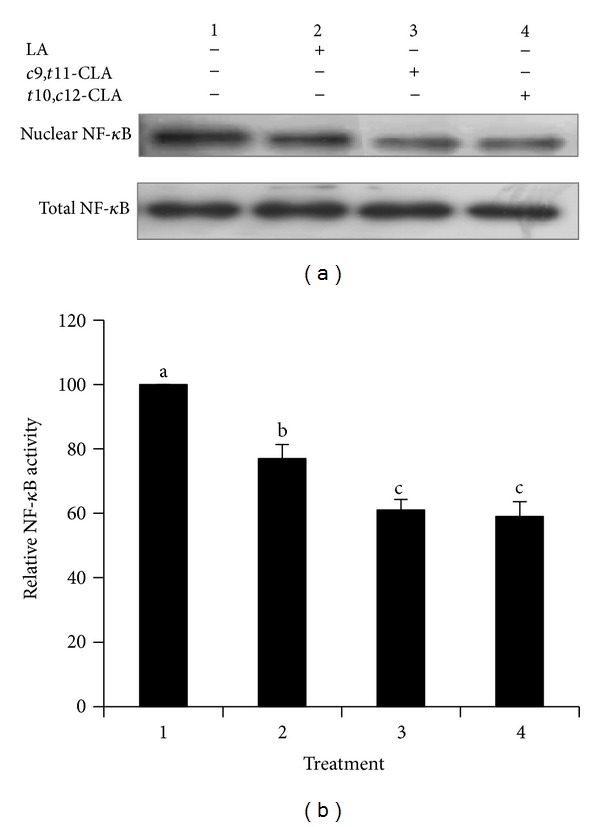
Inhibition of NF-*κ*B activity in MCF-7 cells treated with 40 *μ*M *c*9,*t*11-CLA, *t*10,*c*12-CLA, and LA for 48 h. (a) Western blot analyses of nuclear NF-*κ*B. (b) Band intensities relative to untreated control cells. Values are expressed as means ± standard deviations (*n* = 3). Means with different lowercase letters are significantly different at *P* < 0.05 by Duncan's multiple-range test.

NF-*κ*B is involved in cell proliferation and survival and in the induction of apoptosis, and Cx43 expression is regulated by NF-*κ*B activity [40, 41]. To analyze if the effects of the CLA isomers on inhibited cell growth are related to NF-*κ*B activity, the expression of NF-*κ*B in MCF-7 cells treated with the *c*9,*t*11-CLA and *t*10,*c*12-CLA isomers was determined by Western blotting (Figures 6(a) and 6(b)). The *c*9,*t*11-CLA and *t*10,*c*12-CLA isomers significantly decreased the level of the NF-*κ*B nuclear fraction relative to the control and LA-treated cells. These results indicate that inhibiting NF-*κ*B activity might be involved in the inhibition of MCF-7 cell growth by these CLA isomers.

## 4. Discussion

We showed previously that both *c*9,*t*11-CLA and *t*10,*c*12-CLA isomers inhibit the growth of human MCF-7 breast cancer cells by inducing apoptosis [8]; however, the exact mechanism was not clearly elucidated. It is evident that enhancing GJIC through Cx43 upregulation in tumor cells is positively correlated with anticancer activities by inhibiting the growth of cells through the induction of apoptosis [32, 37, 38, 42, 43]. Hence, the main questions addressed by these studies are whether the growth inhibitory effect of *c*9,*t*11-CLA and *t*10,*c*12-CLA isomers on MCF-7 cells, which reduces GJIC, is mediated by enhanced GJIC to induce apoptosis and whether the effects are associated with upregulation of Cx43.

Apoptosis is one of the most common mechanistic actions of anticarcinogenic CLA isomers in animal and cancer cells [7–9]. The efficacy of individual CLA isomers on the induction of apoptosis is partly mediated by mitochondrial dysfunctional apoptosis, but the mechanism of action is completely unknown. Rakib et al. [13, 29] demonstrated an antipromotional action of *c*9,*t*11-CLA, *t*10,*c*12-CLA, and *t,t*-CLA isomers mediated by the inhibition of downregulation of GJIC in human MCF-10A cells, a nontumorigenic mammary epithelial cell line, treated with TPA, and they suggested that CLA isomers protected phosphorylation of Cx43 to maintain GJIC. These results suggest that CLA isomers could restore the down-regulated GJIC in cancer cells, similar to MCF-7 cells. This hypothesis was solved in the present study as the *c*9,*t*11-CLA and *t*10,*c*12-CLA isomers induced apoptosis by enhancing GJIC through Cx43 gene expression (Figures 3 and 4). Our results agree with reports that anticarcinogens, such as caffeic acid phenethyl ester, coleusin factor, tellimagrandin, and fucoxanthin, induce apoptosis via enhanced GJIC with elevated Cx expression [26, 32, 37, 38].

**Figure 7:**
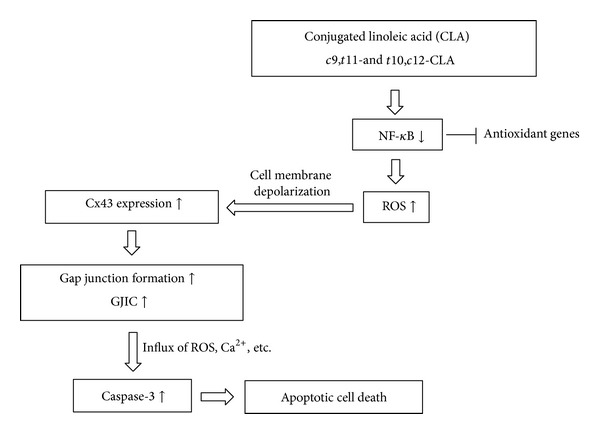
Schematic hypothetical diagram of growth inhibition of MCF-7 cells by apoptosis in relation to enhanced GJIC treated with *c*9,*t*11-CLA and *t*10,*c*12-CLA isomers. Oxidative stress induced by CLA isomers might depolarize the cell membrane and open gap junction channel by expressing Cx43. Open gap junction channels allow exchange of cell death signaling ions and molecules which accelerate cell death through apoptosis as discussed in this study.

ROS are elevated in apoptotic cancer cells induced by CLA isomers [44], whereas NF-*κ*B activation is inhibited [45]. Hence, it is of significance to elucidate that the elevated GJIC through Cx43 protein expression in MCF-7 cells by *c*9,*t*11-CLA and *t*10,*c*12-CLA isomers (Figure 4) is associated with enhanced ROS levels (Figure 5) and/or reduced NF-*κ*B activity (Figure 6). Many chemical compounds that exhibit anticarcinogenic activity can reduce NF-*κ*B activity and elevates ROS level in cancer cells. For example, anticarcinogenic niclosamide reduces NF-*κ*B activity and elevates ROS concentration in acute myelogenous leukemia stem cells [34]. Evidently, the loss of NF-*κ*B results in the accumulation of ROS because NF-*κ*B activity suppresses the expression of some antioxidant genes [46–48]. The elevated ROS, which decreases the fluidity of the biological membranes and increases membrane permeability, depolarizes the cell membrane, opens connexin hemichannels, and expresses connexin genes, resulting in increased cell death [39]. Moreover, the NF-*κ*B inhibitor BAY11-7082 stimulates Cx43 gene expression and restores GJIC in the Toll-like receptor (TLR3) ligand polyinosinic cytidylic acid-induced downregulation of Cx43 gene expression and GJIC [49]. These results suggest that compounds exhibiting anticarcinogenic action produce ROS to induce Cx43 gene expression and enhance GJIC in cancer cells. Taken together, the apoptosis induced by the *c*9,*t*11-CLA and *t*10,*c*12-CLA isomers in MCF-7 cells was associated with upregulation of GJIC through Cx43 expression mediated by inactivation of NF-*κ*B and production of ROS as shown in Figure 7.

One of the known mechanisms of restoring GJIC is the upregulation of connexin gene expression. Down-regulated connexin gene expression in many cancer cells is related to impaired Cx43 expression, including transcriptional silencing through DNA methylation at the connexin gene promoter sites [50]; lower concentrations of cAMP or estrogen [51]; and instability of mRNA and the Cx43 protein [52]. Although further studies are needed to clarify these mechanistic issues involved in the CLA isomers regarding expression of Cx43 in MCF-7 cells, we found that both the *c*9,*t*11-CLA and *t*10,*c*12-CLA isomers clearly reversed the reduced GJIC in MCF-7 cells by upregulating Cx43 genes at the transcriptional and translational levels (Figure 4). This finding was consistent with the result that fucoxanthin [32] and quinoline [53] compounds upregulate GJIC and Cx43 expression in human SK-HP-1 hepatoma cells and human T47D breast cancer cells in relation to growth inhibition. The open hemichannels allow direct entry of intracellular ROS in connexin-expressing cells leading to cancer cell death by altering cell survival mechanisms, including mitochondrial dysfunction [54]. In the present study, increased intracellular ROS were observed in the CLA isomer-treated MCF-7 cells along with increased DNA fragmentation, DNA condensation, and caspase-3 expression (Figure 2). Consequently, the influx of ROS into the cells through the open hemichannels might be involved in the caspase-dependent MCF-7 cell death.

It has been revealed that the antiproliferative activity of individual CLA isomers is dependent upon cancer cells. The anticarcinogenic efficacies of *t*10,*c*12-CLA and *c*9,*t*11-CLA isomers are nearly identical in MG-63 osteosarcoma cells and human MCF-7 breast cancer cells [8, 9], but different efficacies are observed in HT-29 colorectal cancer and PC-3 prostate cancer cells with the stronger potential of *t*10,*c*12-CLA [55, 56]. In the present study, we found no differences in efficacy of *c*9,*t*11-CLA and *t*10,*c*12-CLA to inhibit proliferation of MCF-7 cells with enhanced GJIC by inactivating NF-*κ*B and reducing ROS. These results might be partly due to the similar structural configuration of the *c*9,*t*11-CLA and *t*10,*c*12-CLA isomers. Incorporation of these CLA isomers in the plasma membrane of MCF-7 cells is believed to be one of the factors dictating their similar anticancer activity, including Cx43 gene expression and inactivation of NF-*κ*B, which affect the distribution and function of several membrane raft associated proteins [57, 58]. Furthermore, these CLA isomers may compete as ligands for the NF-*κ*B transcription factor in cancer cells [34], and hence, the similar effects of *c*9,*t*11-CLA and *t*10,*c*12-CLA isomers on inhibiting MCF-7 cell proliferation might be due to similar binding affinities of the CLA isomers to NF-*κ*B expressed in the cells. Further studies are needed to clarify these issues.

In conclusion, both *c*9,*t*11-CLA and *t*10,*c*12-CLA isomers effectively inhibited growth of MCF-7 cells, and this effect was associated with enhanced GJIC through the upregulation of Cx43 expression in conjunction with inactivation of NF-*κ*B. Our results support the hypothesis that CLA isomers upregulate reduced GJIC, which plays a role in propagating cell death as a result of upregulated caspase-3 activity.
